# Assessment of disease activity and quality of life in patients with recurrent bradykinin-mediated versus mast cell-mediated angioedema^☆^

**DOI:** 10.1016/j.waojou.2021.100554

**Published:** 2021-06-16

**Authors:** Pelin Kuteyla Can, Ece Nur Degi̇rmentepe, Piril Etikan, Kübra Kiziltaç, Asli Gelincik, Semra Demir, Suna Buyukozturk, Eda Haşal, Emel Bülbül Başkan, Ömür Aydin, Marcus Maurer, Karsten Weller, Emek Kocaturk

**Affiliations:** a Bahcesehir University, Faculty of Medicine, Department of Dermatology, Istanbul, Turkey; b Okmeydani Training and Research Hospital, Dermatology and Venerology, Istanbul, Turkey; c Istanbul University, Istanbul Faculty of Medicine, Adult Allergy Clinic, Istanbul, Turkey; d Uludağ University Faculty of Medicine Department of Dermatology, Bursa, Turkey; e Ankara University Faculty of Medicine, Adult Allergy Clinic, Ankara, Turkey; f Dermatological Allergology, Allergie-Centrum-Charité, Department of Dermatology and Allergy, Charité - Universitätsmedizin Berlin, Berlin, Germany; g Koç University School of Medicine Department of Dermatology, Istanbul, Turkey

**Keywords:** Angioedema, Angioedema activity score, Angioedema quality of life questionnaire, Bradykinin mediated angioedema, Mast cell mediated angioedema

## Abstract

**Objective:**

Recurrent Angioedema (RAE) is characterized by sudden swelling of mucosal surfaces or deep dermis and is either mast cell-(MMAE) or bradykinin-mediated (BMAE). How patients with BMAE and MMAE differ in terms of disease activity and impact remains largely unknown. Here, we determined validity, reliability, and sensitivity to change of Turkish versions of angioedema activity score (AAS) and quality of life questionnaire (AE-QoL) and used both instruments to investigate and compare patients with BMAE and MMAE.

**Methods:**

Turkish versions of AAS28 and AE-QoL were applied to 94 patients with RAE (18–72 years). Patients’ global self-assessment of QoL (PGA-QoL), disease activity (PGA-DA-VRS, PatGA-DA-VAS), and 12-Item-Short Form Survey were used at week 4 (visit 2), and week 8 (visit 3). Demographic characteristics, clinical features, and AAS28 and AE-QoL values were compared between 31 patients with BMAE and 63 patients with MMAE.

**Results:**

Turkish AAS28 and AE-QoL showed excellent internal consistency, high reproducibility and known-groups validity. Compared to patients with MMAE, BMAE patients were younger (34.6 ± 10.7 vs. 40.7 ± 13.3 years), had longer disease duration (236 ± 178 vs. 51 ± 78 months), high prevalence of family history (63% vs 14%), longer duration of attacks (65 ± 20 vs. 40 ± 25 h), and they were more commonly affected by upper airway angioedema (70% vs 23%). Disease activity (AAS28) was lower (29.3 ± 24.6 vs 55.2 ± 52.9), but AE-QoL was higher (44.2 ± 16.1 vs 34.5 ± 22.5) in BMAE patients as compared to MMAE patients.

**Conclusions:**

Patients with BMAE and MMAE have distinct disease characteristics. Recurrent bradykinin-mediated angioedema impacts quality of life more than mast cell-mediated angioedema. The discriminating characteristics of patients with BMAE and MMAE may help to improve the diagnosis and management of patients with RAE.

## Introduction

Recurrent angioedema (RAE) is the reoccurrence of localized deep dermal, subcutaneous, or submucosal edema resulting from increased vascular permeability and extravasation for longer than 6 weeks. RAE is classified as mast cell-mediated angioedema (MMAE), bradykinin-mediated angioedema (BMAE), and angioedema of unknown origin.^1^, ^2^, ^3^

MMAE can be acute or recurrent, ie, chronic. Acute MMAE occurs in anaphylaxis, with or without wheals,^4^ through an allergic mechanism, specifically type I hypersensitivity, leading to the activation and degranulation of mast cells and their release of mediators such as histamine and leukotrienes.^4^^,^^5^ Acute forms of MMAE can also be caused by nonallergic non-IgE-mediated responses, in response to medications such as non-steroidal anti-inflammatory drugs (NSAIDs) or infections.^6^ Recurrent MMAE most commonly occurs due to chronic urticaria (CU), either chronic spontaneous urticaria (CSU) or chronic inducible urticaria (CIndU), with or without wheals. In case of the latter, MMAE is sometimes referred to as idiopathic histaminergic acquired angioedema.^6^

BMAE occurs due to the increased production of bradykinin or impaired degradation of bradykinin.^4^ Recurrent BMAE subtypes include hereditary angioedema (HAE) and acquired forms.^1^^,^^3^^,^^7^ HAE includes HAE with C1–INH deficiency with low antigenic and functional C1–INH levels (type 1 HAE, HAE-1, 85% of cases), HAE due to C1–INH dysfunction (Type 2 HAE, HAE-2, 10% of cases), characterized by normal (or elevated) antigenic but low functional C1–INH levels, and HAE with normal C1–INH (about 5% of cases). The latter forms of HAE are due to mutations in the F12 gene (HAE-FXII), the angiopoietin-1 gene (HAE-ANGPTI), the plasminogen gene (HAE-PLG), the kininogen1 gene (KNG1), the myoferlin gene (HAE-MYOF), or as of yet unknown mutations (HAE-UNK). Acquired forms of BMAE include angiotensin converting enzyme (ACE) inhibitor-induced AE, AE due to acquired C1–INH deficiency, and acquired idiopathic non-histaminergic AE; while the former is due to reduced catabolism of bradykinin, the latter is due to consumption of C1–INH by the neoplastic lymphatic tissues and/or anti-C1-INH neutralizing autoantibodies.^3^^,^^4^^,^^8^, ^9^, ^10^

Recurrent BMAE is less common and often more severe than recurrent MMAE.^3^^,^^4^ Recurrent BMAE differs from recurrent MMAE in that laryngeal swellings can occur, which can be life threatening, and it does not respond to antihistamine therapy, omalizumab, corticosteroids, or epinephrine treatment.^8^^,^^11^ Instead, C1–INH concentrates, the bradykinin B2-receptor antagonist icatibant, and the kallikrein inhibitors lanadelumab and ecallantide are the drugs of choice in HAE and other types of recurrent BMAE.^3^^,^^12^^,^^13^ The differences in clinical features of the 2 forms of RAE, ie, recurrent MMAE and BMAE, remain ill defined. Whether or not disease activity and impairment of quality of life (QoL) are different in patients with recurrent MMAE and BMAE is currently unknown.

Disease activity, disease impact on QoL, and disease control in patients with RAE are assessed with patient-reported outcome (PRO) measures. These include the Angioedema Activity Score (AAS) and Hereditary Angioedema Activity Score (HAE-AS), the Angioedema Quality of Life Questionnaire (AE-QoL) and Hereditary Angioedema Quality of Life Questionnaire (HAE- QoL), and the Angioedema Control Test.^14^, ^15^, ^16^, ^17^, ^18^, ^19^ The AAS, the AE-QoL and the AECT can be used for both MMAE and BMAE, and these PRO measures are available in many languages and were used in many clinical studies.^15^^,^^20^^,^^21^

This study aims to investigate the differences between patients with recurrent BMAE and MMAE with respect to demographic and clinical characteristics with a focus on disease activity and disease impact on QoL. To this end, we established the validity, reliability and sensitivity to change of the Turkish version of the AAS and the AE-QoL, and we applied both tools to both, patients with recurrent BMAE and recurrent MMAE.

## Materials and methods

### Patients

Patients with RAE (n = 94, 18–72 years, 63% females) from 4 departments (Urticaria Center of Reference and Excellence [UCARE]^22^ and Angioedema Center of Reference and Excellence [ACARE]^23^) were included in this study.

Prerequisites for inclusion in the study were 1) being 18 years old or older, 2) the presence of current RAE, as well as 3) being literate in Turkish. The diagnosis of RAE was based on internationally established criteria – history, clinical symptoms, and allergic status. A positive family history, onset of symptoms in childhood/adolescence, absence of wheals, occurrence of upper airway edema, recurrent and painful abdominal symptoms, and/or failure to respond to antihistamines, systemic steroids or epinephrine, and presence of prodromal symptoms prompted laboratory investigation for bradykinin-mediated angioedema. Diagnosis of HAE type 1 was made if C1 inhibitor antigenic levels were <50% of the normal range and the C1 inhibitor functional levels <50% of the normal range, HAE type 2 if C1 inhibitor antigenic levels were within normal range but the C1 inhibitor functional levels were <50% of the normal range, HAE with mutation in the F12 gene (FXII-HAE) if a mutation was detected in this gene in patients with normal C1 inhibitor antigenic and functional levels. For the diagnosis of RAE due to acquired C1–INH deficiency (AAE), C1 inhibitor antigenic levels of <50% of the normal range and the C1 inhibitor functional levels <50% of the normal range and low C1q levels were required.^3^ Patients were categorized as either BMAE or MMAE according to their treatment responses and laboratory findings.

A total of 94 patients, 31 (33%) patients with recurrent BMAE and 63 (67%) with recurrent MMAE, were included in the study (Table 1). Of the BMAE patients, 26 (83.9%) had HAE type 1, one (3.2%) had HAE type 2, one (3.2%) had FXII-HAE, and three (9.7%) patients had AAE. Of the 63 patients with MMAE, all had chronic spontaneous urticaria (CSU); sixty (95.2%) with wheals and 3 (4.8%) without. Standard doses of second generation antihistamines (in 37 patients, 58.7%), updosed second generation antihistamines (n = 5, 7.9%) and omalizumab (n = 21, 33.3%) were the treatments in MMAE patients, whereas on-demand treatment (C1–INH or icatibant) (n = 12, 38.7%), tranexamic acid (n = 2, 6.5%) and danazol (n = 17, 54.8%) were the treatments received by the BMAE patients.

**Table 1:** Demographic data and scores of patient related outcomes of the bradykinin (BMAE) and mast cell mediated angioedema (MMAE).

n (%)	BMAE	MMAE	p
	n:31, 33%	n:63, 67%	
**Age in years** mean ± sd (range/median) n:94	34.5 ± 10.7 (20–66/34)	40.6 ± 13.3 (18–72/41)	0.028^a^
**Gender** n:94			
female	19 (61.3%)	40 (63.5%)	0.836^b^
male	12 (38.7%)	23 (36.5%)	
**Disease duration in months**^d^ mean ± sd (range/median) n:94	236 ± 178.0 (12–720/200)	51.4 ± 78.2 (3–360/24)	p < 0.001^a^
**Duration of attack in hours**^e^ mean ± sd (range/median) n:61	65.0 ± 19,7 (24–120/72)	39.5 ± 24.9 (0–72/24)	p < 0.001^a^
**Positive family history for RAE** n:92	19 (63.3%)	9 (14.5%)	p < 0.001^b^
**Triggering factors for RAE** n:94	26 (83.9%)	50 (79.4%)	0.602^b^
stress	17 (65.4%)	37 (74%)	
trauma	7 (26.9%)	1 (2%)	
tiredness	1 (3.8%)	0	
cold air	1 (3.8%)	1 (2%)	
others	0	11 (22%)	
**Location of RAE,** n:61			
lips	30 (100%)	31 (100%)	–
periorbital region	29 (96.7%)	28 (90.3%)	0.612^c^
hands	22 (73.3%)	12 (38.7%)	0.006^b^
feet	19 (63.3%)	5 (16.1%)	p < 0.001^b^
genital	2 (6.7%)	4 (12.9%)	0.671^c^
**Presence of upper airway AE** n:61	21 (70.0%)	7 (22.6%)	p < 0.001^b^
**AE-QoL at week 4 (visit 2)** mean ± sd (range/median) n:94	44.2 ± 16.1 (14.7–75/47.0)	34.5 ± 22.5 (0–83.8/33.8)	0.029^a^
**AE-QoL at week 8 (visit 3)** mean ± sd (range/median) n:60	40.4 ± 17.9 (4.4–75/40.4)	32.7 ± 22.4 (0–72/32.3)	0.153^a^
**SF-12 Physical composite summary (PCS) at week 4 (visit 2)** mean ± sd (range/median) n:61	40.7 ± 6.9 (29.8–54.9/41.0)	43.6 ± 5.8 (30.9–57.2/43.2)	0.065^a^
**SF-12 Physical composite summary (PCS) at week 8 (visit 3)** mean ± sd (range/median) n:60	42.42 ± 6.03 (28.8–52.6/43.4)	45.9 ± 5.5 (32.8–55.1/46.2)	0.025^a^
**SF-12 Mental composite summary (MCS) at week 4 (visit 2)** mean ± sd (range/median) n:61	44.6 ± 5.9 (32.8–58.3/44.6)	43.8 ± 6.7 (25.1–57.7/43.9)	0.937^a^
**SF-12 Mental composite summary (MCS) at week 8 (visit 3)** mean ± sd (range/median) n:60	45.6 ± 5.3 (34.9–59.0/46.3)	44.5 ± 5.6 (33.2–53.7/44.4)	0.554^a^
**AAS28 at week 4 (visit 2)** mean ± sd (range/median) n:94	29.3 ± 24.6 (0–122/23)	55.2 ± 52.9 (0–242/42)	0.011^a^
**AAS28 at week 8 (visit 3)** mean ± sd (range/median) n:93	21.7 ± 14.2 (0–52/22)	28.6 ± 35.7 (0–155/15.5)	0.554^a^
**PatGA-DA-VAS at week 4 (visit 2)** mean ± sd (range/median) n:61	48.8 ± 29.9 (0–85/50)	41.1 ± 28.1 (0–85/40)	0.276^a^
**PatGA-DA-VAS at week 8 (visit 3)** mean ± sd (range/median) n:60	41.0 ± 26.9 (0–85/40)	26.3 ± 26.1 (0–80/17.5)	0.031^a^
**Number of days (last 4 weeks) affected by RAE at week 4**^f^ mean ± sd (range/median) n:61	3.8 ± 3.4 (0–17/3.5)	6.7 ± 6.8 (0–28/4)	0.09^a^
**Number of days (last 4 weeks) affected by RAE at week 8** mean ± sd (range/median) n:60	3.3 ± 2.5 (0–10/3)	3.9 ± 2.5 (0–17/2)	0.607^a^

Angioedema activity score (AAS), angioedema quality of life questionnaire (AE−QoL), bradykinin cell mediated angioedema (BMAE), mast cell mediated angioedema (MMAE), patient's global assessment of disease activity-visual analog scale (PatGA−DA-VAS), recurrent angioedema (RAE), standard deviation (sd), the 12−item short form health survey (SF−12)

^a^ p values assesed by Mann Whitney *U* test.

^b^ p values assesed by Pearson Chi-Square test.

^c^ p values assesed by Fisher's exact test.

^d^ Duration of the disease since its first onset.

^e^ Hours that attacks last.

^f^ Reported number of days affected by AE in last 4 weeks

The study was approved by the institutional ethics committee and was conducted according to the Declaration of Helsinki. Written informed consent was obtained from all participating patients.

### Translation and linguistic validation of the Turkish AAS and AE-QoL

Forward and backward translations were performed to obtain validated Turkish versions of both the AAS and AE-QoL. The original German versions of both measures were translated into Turkish, and Turkish versions were reviewed by 2 dermatologists. A Turkish consensus version was developed and back-translated into German by 2 independent bilingual translators. The back-translated versions were then reviewed by the original authors. Potential misconceptions or misinterpretations introduced in the translation process were discussed between the Turkish research team and the original authors. After a consensus meeting, the final versions of the questionnaires were tested for cognitive debriefing in a pilot group of 10 patients with RAE.

### Patient reported outcome measures and clinical assessment

The AAS is a diary-type tool in which the patients are asked to record their angioedema symptoms once daily. The AAS consists of 5 items (AE duration, physical discomfort caused by AE, impact of AE on daily activities, impact of AE on appearance, AE overall severity) that are each scored from 0 to 3 each day, resulting in daily scores of 0–15 points). Daily AAS scores can be summed up to 7-day scores (AAS7, 0–105 points), 4-week scores (AAS28, 0–420 points), 8-week scores (AAS56, 0–840 points) and 12-week scores (AAS84, 0–1260 points). The minimally clinically important difference (MCID) of the AAS7 was found to be 8 points.^16^

The AE-QoL is a disease-specific QoL questionnaire with 17 questions. Each item (question) scores between 0 and 4 points, and a raw total score as well as 4 raw different domain scores (functioning, fatigue/mood, fears/shame, and food) may be computed. All scores are then transformed into a linear scale that ranges from 0 to 100, with a score of 100 indicating the worst possible impairment of health-related quality of life (HRQoL).^17^ The MCID of the AE-QoL total score was found to be 6 points.^24^

**Fig. 1:**
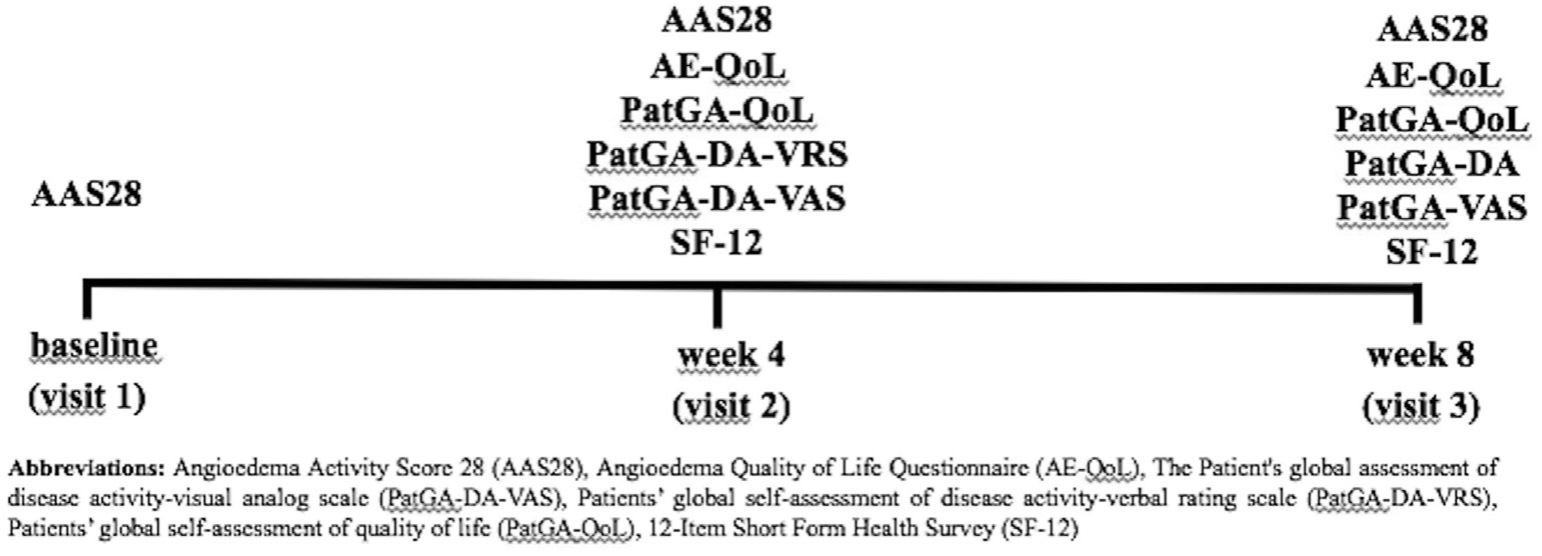
Flow chart of the study

Anchor instruments used to evaluate disease activity as well as AE-related QoL impairment included two patients' global self-assessments of disease activity (PatGA-DA-VRS);^16^^,^^25^ PatGA-DA-VAS,^26^ a patients’ global self-assessment of QoL impairment (PatGA-QoL),^24^ and the 12-Item Short Form Health Survey (SF-12).^27^ The AAS28 (angioedema activity score of 28 days) was provided to all patients at the baseline visit (Visit 1) for the duration of the study. At baseline (Visit 1), week 4 (Visit 2), and week 8 (Visit 3), all patients completed the AE-QoL, PatGA-DA-VRS, PatGA-DA-VAS, PatGA-QoL, and SF-12 (Fig. 1).

The PatGA-DA-VRS is a self-administered verbal rating scale that assesses disease activity during the past 4 weeks on a 4-point scale: 0 = absent, 1 = mild, 2 = moderate, and 3 = severe.^16^^,^^25^

The PatGA-DA-VAS is a self-administered visual analogue scale that assesses disease activity during the past 4 weeks. Patients specify their complaint level by indicating a position along a continuous unmarked line with the endpoints “no complaints” (0 cm) and “maximal complaints” (10 cm).^26^

The patients’ global self-assessment of the effect of RAE on HRQoL impairment (PatGA-QoL) uses one question on the past 4 weeks period with a 4-point Likert-scale: 0 = no effect, 1 = mild effect, 2 = moderate effect, 3 = severe effect.^24^ 

The 12-Item Short Form Health Survey (SF-12) contains 12 questions and is a validated short version of the SF-36. It has a Physical Composite Summary (PCS) and a Mental Composite Summary (MCS) ranging from 0 to 100 points. Higher scores indicate a better level of health.^27^

Age, gender, disease duration, duration of RAE attacks, family history, number of days affected by RAE (NDAE), triggers, site of RAE, and accompanying upper airway AE were recorded.

### Assessment of the Turkish AAS and AE-QoL validity, reliability, and screening accuracy

#### Internal consistency

Cronbach's α coefficient was computed to determine the internal consistency reliability of the Turkish AAS28 and AE-QoL. The Cronbach's α coefficient was interpreted as follows: <0.60 unacceptable, 0.60–0.65 undesirable, 0.65–0.70 minimally acceptable, 0.70–0.80 respectable, 0.80–0.90, excellent and >0.90 excessive consistency.^25^

#### Convergent validity

In order to determine the convergent validity of the Turkish AAS28 and AE-QoL, their results were correlated to the PatGA-DA-VRS, the PatGA-DA-VAS, and the PatGA-QoL by computing the Spearman's rho correlation coefficient. Weak, moderate, and strong correlations were defined by correlation coefficient values of <0.3, 0.3–0.5, and >0.5, respectively.^24^^,^^28^

#### Known-groups validity

Known-groups validity assesses the ability of a tool to discriminate between patients with different disease states. To determine known-groups validity, we compared the mean AAS28 and AEQoL scores of patients with different levels of disease activity (PatGA-DA-VRS) and HRQoL impairment (PatGA-QoL) by using the Kruskal-Wallis test.

#### Sensitivity to change

AAS28 and AE-QoL data was available for 93 and 60 patients at the third visit, respectively (Table 1). To assess sensitivity to change, the changes in the AAS28, AE-QoL scores and SF12, PatGA-DA-VRS, PatGA-DA-VAS, and the PatGA-QoL scores from the second to the third visit were calculated and the correlation between the score changes was analyzed by computing Spearman's rho.

#### Test-retest reliability (intraclass correlation coefficient - ICC)

To determine the test-retest reliability of the Turkish AAS28 and AE-QoL, only patients who had no change in the PatGA-DA-VRS scores for AAS28 and PatGA-QoL for AE-QoL during the four-week interval between visit 2 and 3 (as a surrogate for stable disease activity and stable QoL impairment) were included to calculate the intraclass correlation coefficient. An ICC of 0.5–0.7 indicated moderate-to-good reproducibility while an ICC of greater than 0.7 was considered to demonstrate excellent reproducibility.^25^

#### Screening accuracy (categorization)

Screening accuracy (categorization) is the ability of the AAS28 to categorize patients into having meaningful disease activity versus non-meaningful disease activity and the ability of the AE-QoL to categorize patients into suffering from meaningfully impaired HRQoL vs non-meaningfully impaired HRQoL. ROC curve analyses were used to determine screening accuracy of the AAS28 and AE-QoL. To this end, PatGA-DA-VRS results were categorized into meaningful disease activity (moderate, severe, n:35) and non-meaningful disease activity (none and mild, n:26) and PatGA-QoL was categorized into patients with meaningful impaired HRQoL (severe and moderate effect, n:35) and non-meaningful impaired HRQoL (mild and no effect, n:26). The cut off values for meaningful vs non-meaningful disease activity, and meaningful impaired vs non-meaningful impaired HRQoL were determined for visit 2. An area under the curve (AUC) of 1, 0.9, 0.8, 0.7, and 0.5 were interpreted as perfect, excellent, good, fair, and no better than chance, respectively.^29^

### Statistical analysis

All statistical analyses were performed using SPSS (IBM SPSS Statistics version 22). The statistical methods for validation are described at the respective methods section of this manuscript. All numerical variables were reported as the mean ± standard deviation (SD), median, minimum, maximum, frequency and percentages. Differences in measured parameters between the BMAE or MMAE groups were analyzed with Mann–Whitney *U* test (age, disease duration, duration of attack, mean scores of AAS28, AE-QoL, PatGA-VAS, Physical Composite Summary (PCS), and a Mental Composite Summary (MCS) of SF12, number of days affected, items of AAS28 and domains of AE-QoL). The comparison of the qualitative variables such as gender, family history, triggers, side of RAE, accompanying laryngeal edema of BMAE or MMAE groups was analyzed with Pearson's chi-square test, or Fisher's Exact test. A p-value of <0.05 was regarded as statistically significant.

## Results

### The Turkish AAS28 and AE-QoL are valid and reliable instruments to evaluate disease activity and QoL in patients with RAE

The Turkish AAS28 showed high levels of internal consistency, with a Cronbach's α of 0.964. The ICC was used to demonstrate test-retest-reliability, and the ICC for the AAS28 (0.693) indicated good reproducibility (Table 2). The Turkish AE-QoL total score showed high levels of internal consistency, with a Cronbach's α of 0.925. Also, all individual domains of the AE-QoL, except the nutrition domain, were found to have excellent internal consistency (Table 2). The ICC for the AEQoL total score (0.882) and the domain scores (0.747–0.827) demonstrated excellent reproducibility (Table 2).

**Table 2:** Internal consistency and test-retest reliability of the Turkish AAS28 and AEQoL.

		mean ± sd	Cronbach's Alpha	ICC
**AAS28 week 4, n:61**		46.6 ± 47.0	0.964	**AAS28 n:26**^b^ 0.693
	**Item questions**	**mean** ± **sd**	**Cronbach's Alpha**	**ICC**
**AE-QoL week 4, n:94**				**AE-QoL n:23**^a^
Total	Q1-17	37.7 ± 21.0	0.925	0.882
Functioning	Q1-4	28.8 ± 24.0	0.886	0.827
Fatigue/mood	Q6-10	38.1 ± 25.0	0.880	0.940
Fears/shame	Q12-17	47.2 ± 27.7	0.901	0.805
Nutrition	Q5/11	25.9 ± 26.4	0.548	0.747

Angioedema activity score (AAS), angioedema quality of life questionnaire (AE−QoL), intraclass correlation coefficient (ICC)

^a^ ICC values reflect the analysis of data from 23 patients who had stable PatGA-QoL (no change in PatGA-QoL during the study period [between week 4 and week 8].

^b^ ICC value reflects the analysis of data from 26 patients who had stable PatGA-DA (no change in PatGA-DA during the study period [between week 4 and week 8]

The AAS28 scores showed strong correlations with the number of days affected by RAE (r = 0.844; p < 0.001) and strong correlations with the PatGA-DA-VRS (r = 0.59; p < 0.001) and the PatGA-DA-VAS (r = 0.66; p < 0,001). The AE-QoL showed a significant, albeit weak to moderate correlation with the AAS28 (r = 0.33; p = 0.001) and moderate correlations with the PatGA-QoL (r = 0.48; p < 0.001), PatGA-DA-VRS (r = 0.47; p < 0.001) and PatGA-DA-VAS (r = 0.43; p = 0.001). In addition, the AE-QoL was found to have a weak to moderate correlation with the PCS of the SF-12 (r = −0.31; p = 0.013), but no significant correlation with the MCS of the SF-12 (−0.16; p = 0.214). Taken together, the results indicate good levels of convergent validity for the AAS28 and sufficient levels of convergent validity for the AE-QoL.

The AAS28 was able to discriminate between groups with different levels of disease activity (PatGA-DA-VRS) and different levels of QoL impairment (PhyGA-QoL), ie, patients with higher self-rated disease activity and QoL impairment had higher AAS28 scores and higher AE-QoL values, respectively (Table 3), thus demonstrating known-groups validity of both tools.

**Table 3:** Known groups validity of the AAS28 and AE-QoL.

AAS28	n	mean ± sd (median)	min-max	Interquartile range (lower–upper quartile)
**PatGA-DA-VRS**				
none	5	1.2 ± 2.7 (0)	0–6	0–3
mild	21	20.9 ± 14 (19)	2–48	9–33
moderate	25	46 ± 31.3 (38)	7–122	22–57
severe	10	73 ± 79.2 (38.5)	18–242	95.5–30.7
*p*		<0.001		
**PatGA-QoL**				
no effect	8	4.7 ± 6.6 (2.5)	0–19	0–7.5
mild effect	18	24.4 ± 14.3 (26)	2–48	10.7–38.5
moderate effect	25	51.9 ± 43.8 (38)	7–199	22–66.5
severe effect	10	55.1 ± 67.5 (36)	8–242	20.2–54.2
*p*		<0.001		
**AE-QoL**				
**PatGA-DA-VRS**				
none	5	10.6 ± 8.9 (13.2)	1.5–22	1.5–18.4
mild	21	36.2 ± 19.7 (36.8)	4.4–72	16.9–48.5
moderate	25	48.9 ± 13.9 (48.5)	25–67.6	36.8–61.8
severe	10	50.3 ± 13.6 (50.7)	23.5–75	43.4–57.7
*p*		0.001		
**PatGA-QoL**				
no effect	8	17.6 ± 14.6 (15.4)	1.5–47	4.4–24.3
mild effect	18	37.4 ± 20.5 (36.8)	4.4–72	17.3–52.6
moderate effect	25	48.4 ± 13.8 (47.0)	25–67.6	36.8–61.8
severe effect	10	51.5 ± 13.6 (55.1)	23.5–75	43.4–57.7
*p*		0.001		

All p values were assesed by using the Kruskal Wallis test.

Angioedema activity score (AAS), angioedema quality of life questionnaire (AE−QoL), patients' global self-assessment of disease activity-verbal rating scale (PatGA−DA-VRS), patients' global self-assessment of quality of life (PatGA−QoL)

Mean changes in scores of all patients were included in sensitivity to change analyses. The AAS28 and AE-QoL were sensitive to detect changes in disease activity and HRQoL impairment, respectively. AAS28 changes strongly correlated with PatGA-DA-VAS changes (r = 0.73; p < 0.001), changes in the number of days affected by RAE (r = 0.65; p < 0.001), and changes in PatGA-DA-VRS (r = 0.56; p < 0.001) and PatGA-QoL (r = 0.56; p < 0.001). Changes in AE-QoL total scores showed low to moderate correlations with PatGA-DA-VAS changes (r = 0.36; p = 0,005), PatGA-DA-VRS changes (r = 0.30, p = 0.019), PatGA-QoL changes (r = 0.34; p = 0.007) and weak, albeit significant correlations with changes in the number of days affected by RAE (r = 0.284; p = 0.028) and changes in the MCS of the SF-12 (r = −0.27; p = 0.035).

Thirty-five patients had moderate to severe disease activity RAE based on PatGA-DA-VRS and moderate to severe impairment in QoL based on the PatGA-QoL at the 2nd visit. The performance of the AAS28 and AE-QoL at various cutoff values in screening for patients with meaningful (moderate to severe) disease activity (n:35) and meaningful (moderate to severe) impaired QoL (n:35) is shown in Table 4. Twenty-six patients had no and mild disease activity RAE based on PatGA-DA-VRS and no to mild impairment in QoL based on the PatGA-QoL at the 2nd visit.

**Table 4:** Performance of the AAS28 and AE-QoL at various cutoff values in screening for patients with meaningful disease activity and meaningful impaired QoL. As anchors for meaningful disease activity and impaired QoL, the PatGA-DA-VRS and the PAT-GA-QoL were used respectively.

*Turkish AAS28 score*	*Meaningful disease activity (n:35*^a^*)*		*Turkish AE-QoL*	*Meaningful impaired QoL (n:35*^b^*)*	
*Cutoff value*	Sensitivity (%)	Specificity (%)	*Cutoff value*	Sensitivity (%)	Specificity (%)
*7*	97	69	*24*	97	46
*9*	97	38	*26*	*94*	*50*
*10*	97	46	*29*	*89*	50
*11*	97	50	*32*	*86*	*50*
*14*	94	54	*33*	*86*	*54*
*16*	91	58	*35*	*83*	*58*
*17*	89	58	*37*	*77*	*65*
*18*	83	58	*39*	*74*	*65*
*20*	83	62	*40*	*74*	*69*
*21*	80	65	*42*	*71*	*69*
*22*	74	65	*43*	*69*	*69*
*23*	71	65	*46*	*60*	*69*
*24*	69	65	*48*	*51*	*77*
*25*	69	69	*49*	*49*	*81*
*26*	66	69	*51*	*49*	*85*
*28*	66	77	*54*	*43*	*85*
*32*	66	81	*56*	*40*	*85*
*34*	63	81	*58*	*31*	*85*
*35*	60	81	*59*	*23*	*85*
*37*	51	85	*62*	*20*	*88*
*38*	49	88	*AUC (95% CI)*	0.753 (62–89%)	
*39*	46	88	*-*	*-*	*-*
*40*	46	92	*-*	*-*	*-*
*41*	43	92	*-*	*-*	*-*
*42*	43	96	*-*	*-*	*-*
*44*	40	96	*-*	*-*	*-*
*46*	37	96	*-*	*-*	*-*
*50*	37	100	*-*	*-*	*-*
*AUC (95% CI)*	0.821 (72–92.5%)		*-*	*-*	*-*

Angioedema activity score (AAS), angioedema quality of life questionnaire (AE−QoL), area under the curve (95% confidence interval) (AUC (95% CI)), patients' global self-assessment of disease activity-verbal rating scale (PatGA−DA-VRS), patients' global self-assessment of quality of life (PatGA−QoL)

^a^ Thirty-five patients had moderate to severe disease activity and 26 patients had no and mild disease activity based on PatGA-DA-VRS.

^b^ Thirty-five patients had moderate to severe impairment in QoL and 26 patients had no to mild impairment in QoL based on the PatGA-QoL

### Patients with BMAE differ from patients with MMAE in important clinical features of their disease

Patients with BMAE were significantly younger, their disease duration was markedly longer, and they had a higher rate of other family members affected by RAE as compared to patients with MMAE. All of the BMAE and MMAE patients reported that they had experienced lip swellings (100%), and most had experienced periorbital swellings (BMAE 96.7% vs MMAE 90.3%). Swellings of the hands and feet were more common in BMAE patients (73% and 63% of patients) than in MMAE patients (39% and 16%) (Table 1). Angioedema of the upper airways was reported in 70% (n = 21) of patients in the BMAE group, as compared to 23% (n = 7) of MMAE patients. Across all sites affected by RAE, swelling attacks in BMAE patients were of markedly longer duration as compared to those in patients with MMAE (Table 1). The rate of patients with AE-triggering factors was similar between the two groups. The most common trigger of swellings was stress, in both groups (in BMAE; 65% vs in MMAE; 74%). Trauma was reported as a trigger by 26.9% of the BMAE patients but only 2% of the MMAE patients (Table 1).

### Patients with recurrent BMAE show lower disease activity but higher QoL impairment than patients with recurrent MMAE

The mean AAS28 score in patients with recurrent BMAE was significantly lower as compared to recurrent MMAE patients (p = 0.011). Furthermore, the number of days affected by angioedema was lower in the BMAE group, albeit not statistically significantly (3.8 ± 3.4 vs 6.7 ± 6.8 days per month; p = 0.09) (Table 1).

HRQoL was more impaired in patients with recurrent BMAE, with higher AE-QoL total scores at visit 2 as compared to patients with recurrent MMAE (p = 0.029) (Table 1). This was due to higher impairment in the AE-QoL domains functioning and fear/shame in the BMAE group as compared to the MMAE group (functioning: p = 0.005; fear/shame: p = 0.01) (Table 5).

**Table 5:** Comparison of mean scores of AAS28 items as well as AE-QoL items and domains between bradykinin (BMAE) and mast cell mediated angioedema (MMAE).

2^nd^ visit (week 4)	BMAE	MMAE	*p*
n of patients	n: 31 (33%) mean ± sd (range/median)	n: 63 (67%) mean ± sd (range/median)	
**AAS28 item scores n:61**			
Duration^a^	4.6 ± 4.1 (0–19/4)	7.9 ± 7.6 (0–30/4)	0.187
Physical discomfort caused	6.6 ± 5.7 (0–27/5)	12.3 ± 14.4 (0–63/7)	0.098
Impact on daily activities	4.8 ± 4.7 (0–22/4)	6.0 ± 7.4 (0–28/3)	0.856
Impact on appearance	5.8 ± 5.5 (0–27/4.5)	10.5 ± 13.6 (0–63/6)	0.156
Overall severity	6.8 ± 5.8 (0–28/5.5)	10.9 ± 12.9 (0–58/6)	0.329
**Domains of AE-QoL n:94**			
**Functioning**	38.3 ± 21.5 (0–75/43.7)	24.1 ± 23.8 (0–93.75/18.7)	0.005
Impairment of work	1.6 ± 1.1 (0–4/2)	1.0 ± 1.1 (0–4/1)	0.01
Impairment of physical activity	1.6 ± 1.0 (0–3/2)	1.0 ± 1.1 (0–4/1)	0.007
Impairment of spare time activities	1.2 ± 1.0 (0–3/1)	0.7 ± 1.0 (0–4/0)	0.033
Impairment of social relations	1.7 ± 1.1 (0–3/2)	1.1 ± 1.2 (0–3/1)	0.012
**Fear/shame**	57.1 ± 19.5 (20.8–87.5/62.5)	42.3 ± 29.9 (0–100/41.7)	0.01
Feeling burdened at having swellings	2.2 ± 1.1 (0–4/2)	1.7 ± 1.3 (0–4/2)	0.1
Fear of new suddenly appearing swellings	2.9 ± 0.9 (1–4/3)	1.9 ± 1.5 (0–4/2)	0.002
Fear of increased frequency of swellings	2.7 ± 1.0 (1–4/3)	2.1 ± 1.4 (0–4/2)	0.049
Ashamed to visit public places	1.9 ± 1.3 (0–4/2)	1.3 ± 1.4 (0–4/1)	0.028
Embarrassed by the appearance of swellings	1.7 ± 1.1 (0–4/2)	1.3 ± 1.4 (0–4/1)	0.06
Fear of long term negative drug effects	2.2 ± 1.3 (0–4/2)	1.8 ± 1.5 (0–4/2)	0.149
**Fatigue/mood**	41.4 ± 21.8 (5–80/40)	36.42 ± 26.4 (0–95/35)	0.304
Difficulties of falling asleep	1.5 ± 1.0 (0–4/2)	1.3 ± 1.2 (0–4/1)	0.420
Waking up during the night	1.7 ± 1.1 (0–4/2)	1.6 ± 1.3 (0–4/2)	0.741
Feeling tired during the day	1.7 ± 1.0 (0–4/2)	1.6 ± 1.3 (0–4/1)	0.608
Difficulties in concentrating	1.7 ± 1.1 (0–4/2)	1.3 ± 1.2 (0–4/1)	0.106
Feeling downhearted	1.7 ± 1.2 (0–4/2)	1.4 ± .1.3 (0–4/1)	0.225
**Nutrition**	24.2 ± 23.5 (0–75/25)	26.8 ± 27.8 (0–100/25)	0.865
General limitations in foods and eating	1 ± 1.2 (0–4/0)	0.8 ± 1.1 (0–4/0)	0.430
Limitations in the selection of food and beverages	0.9 ± 1.3 (0–4/0)	1.3 ± 1.4 (0–4/1)	0.128

All p values were assesed by Mann Whitney *U* test.

Angioedema activity score (AAS), angioedema quality of life questionnaire (AE-QoL), bradykinin cell mediated angioedema (BMAE), mast cell mediated angioedema (MMAE).

^a^ Swelling duration was scored according to the time period that patients experience the swelling such as; midnight – 8 a.m./8 a.m.–4 p.m./4 p.m. – midnight; each represents 1 points

## Discussion

Our results show that the 2 subtypes of RAE have distinctive clinical characteristics and a substantial impact on patients' QoL. To our knowledge this is the first study to compare the impairment caused by these 2 diseases on different aspects of daily life and also disease activity. RAE has a fluctuating disease activity and can vary from day to day, and it can lead to life-threatening episodes and have substantial effect on patients’ HRQoL.^15^ The AAS and AE-QoL are reliable tools to assess the disease activity and HRQoL and versions of both PROMs are available in many languages.^21^

Family history is very important in patients with isolated RAE. If positive, a diagnosis of HAE can be confirmed.^7^ As expected, a positive family history was more prevalent in patients with BMAE. In a study comparing the clinical features of HAE and MMAE, the incidence of familial AE was reported as 73.9% in the HAE group which was significantly higher than that in the MMAE group (9.7%).^28^ We are not sure if this frequency of family history in the MMAE group really points to a true family history of CU since the answers might be attributed to acute urticaria as well as other itching conditions which have been perceived as CU.

BMAE develops gradually, builds within hours or days, and can last 3–5 days, while MMAE starts within minutes to hours and does not persist longer than 24–48 hours.^6^^,^^8^ Mild trauma and iatrogenic trauma, emotional stress, pregnancy, infections, and estrogen therapy are AE triggering factors that have been identified in HAE. Trauma (11.5%) and emotional stress (32.7%) proved to be the most frequent triggers of symptoms in patients with HAE.^8^^,^^30^, ^31^ Increased levels of stress also may evoke or aggravate symptoms in patients with CSU,^32^^,^^33^ and the most common trigger factor in our study was stress in both groups (in BMAE; 65% vs in MMAE; 74%), and trauma was reported as a trigger by 26.9% of the BMAE patients which was only reported by one of the patients in the MMAE group.

In all subtypes, most RAE attacks occur in the face and in the oropharynx.^5^ Oropharyngeal involvement is more commonly seen in BMAE.^34^ In our study, the upper airway was found to be involved in 70% of the patients with BMAE, while 23% in the MMAE group. Our results are generally in line with previous reports. However, our results depend on patients’ self-reports, and we do not know for sure which parts of the upper airway were affected exactly, if a laryngeal AE was present or whether the AE was associated with upper airway obstruction (choking) or not.

This study includes validation of the AAS and AE-QoL to Turkish which showed excessive internal consistency (Cronbach's α Asian versus Turkish version AAS28 0.97 vs 0.96, original German versus Turkish version AE-QoL 0.89 vs 0.93).^17^^,^^35^ AAS28 indicated good reproducibility and both AE-QoL total score and domains scores demonstrated excellent reproducibility. As expected the AAS28 showed strong correlations with anchors for disease activity and the AE-QoL total scores showed moderate correlations with PatGA-QoL, indicating sufficient levels of convergent validity for the AAS28 and the AE-QoL. In addition, AAS28 changes strongly correlated with changes in the anchors for disease activity and AE-QoL total score changes moderately correlated with changes in PatGA-QoL, but only weakly, with changes in the MCS of the SF12. Weller et al. also showed weak correlations between AE-QoL score changes and changes in the MCS and PCS of the SF-12 and moderate correlations with PatGA-DA-VRS and PatGA-QoL changes just as in our study.^24^ The most likely reason for this is that the SF-12 is a generic and not angioedema-specific tool, and, therefore, is less sensitive to changes. Probably, the dermatology life quality index (DLQI) would be more suitable than the SF-12 because the DLQI was specifically designed for disorders affecting the skin.^36^ In the Thai validation study, AE-QoL total scores higher than 38 and 36 were reported to indicate a ‘moderate to large effect’ on HRQoL.^28^ Our results suggest an AE-QoL total score of 40 to be a good cutoff value to identify patients with a meaningfully impaired HRQoL, since this cutoff value shows a good balance of sensitivity and specificity. For the AAS28, the results on the most suitable cutoff value are less clear and suggest a cutoff between 20 and 35 points.

The average attack number per year was reported to be 27 for HAE while it was reported to be 19 in the ASSURE study for patients with CSU.^37^^,^^38^ In the present study, the MMAE group, even though not statistically significant, had more days with angioedema (6.7 vs 3.7) which contrasted with the aforementioned studies.

BMAE tends to be more severe than MMAE and has a substantial impact on QoL.^34^^,^^39^ Nordenfelt et al^39^ reported that the mean AAS28 score was 18.2 among men with HAE and 16.4 among women with HAE and they showed that AAS28 correlated strongly with the functioning domain of the AE-QoL, and the AE-QoL total score in patients with HAE that confirmed that severity of RAE attacks has a negative impact on AE-QoL.^39^ The mean AAS28 was 29.32 in our study, which was much higher than Nordenfelt et al^39^ reported, and we also showed that AAS28 moderately correlates with the AE-QoL total score. In the current study, the mean AAS28 score of the BMAE group was significantly lower while the mean AE-QoL total score was higher than the MMAE group. This seems contradictory, but this shows that the impact of the disease does not depend only on the activity of the disease or frequency of the attacks but on the impact of the disease on the overall social, emotional, and physical well-being that is not only present during an attack but also in between attacks. In our study, HRQoL is impaired significantly more in patients with BMAE; especially, functioning including work, physical activity, spare time activities, social relations and fears/shame (including the fear of suddenly appearing swellings, increased frequency of swellings, and ashamed to visit public places) were significantly more impaired in patients with BMAE than MMAE.

AE is, however, also a major driver of QoL impairment in patients with CSU. RAE causes disfigurement and/or functional impairment, and has significant impact on daily activities and social interactions as well as work and school life.^11^ In the X-ACT study, AE-QoL total scores (omalizumab 300 mg: 56.2; placebo: 59.9) showed a severe HRQoL impairment in patients with RAE associated with CSU. The most severely affected subdomains reported were fears/shame, fatigue/mood, and functioning, while in our study, the domain that was less affected was functioning in MMAE group and the MMAE group had lower scores (both total scores and each domain scores) than reported in the X-ACT study. This might be due to the fact that some of the patients of our study were already under treatment. In the X-ACT study, 59.1% of the patients with RAE in the context of CSU mentioned that they are scared of developing a life-threatening episode.^11^ In HAE patients, RAE can be life-threatening when laryngeal edema occurs (30%), but RAE is considered to be usually not life threatening in CSU.^11^ We showed that the fears/shame domain scored significantly higher in the BMAE group as compared to the MMAE group.

HAE significantly impacts the ability of a patient to work or go to school and patients with HAE are more likely to suffer symptoms of depression than the general population.^40^ We found that mood and functioning were affected more in BMAE than MMAE.

Our study has several strengths and limitations. First of all, we did not link disease activity and impact to treatment. There were a lot more patients with MMAE who received prophylactic treatment than patients with BMAE. This might have exerted an effect on average disease activity and impact. Second, we did not ask for the presence of abdominal attacks, which is a very prominent feature of BMAE. Upper air way involvement was not evaluated by examination at time of the attack which leads to inadequate information on laryngeal attacks.

As a conclusion, there are distinctive features between bradykinin-mediated and mast-cell mediated angioedema, ie, patients with BMAE have younger age and longer disease and attack duration, a higher frequency of positive family history, hand–feet involvement and upper airway edema, and the burden of disease seems to be higher in the BMAE group as compared to the MMAE group, even though the activity of the disease and days with angioedema are lower in the BMAE group. Being aware of the distinguishing features of these 2 major types of RAE and assessing disease activity and impact with the recommended tools may help to aid a better diagnostic and therapeutic management of RAE patients.

## Abbreviations

Angioedema Activity Score; (AAS), Angioedema Centers of Reference and Excellence; (ACARE), Angioedema Control test; (ACT), Angioedema quality of life questionnaire; (AE-QoL), area under the curve; (AUC), bradykinin-mediated angioedema; (BMAE), dermatology life quality index; (DLQI), health-related quality of life; (HRQoL), Hereditary Angioedema Activity Score; (HAE-AS), Hereditary Angioedema quality of life questionnaire; (HAE-QoL), intraclass correlation coefficient; (ICC), mast cell mediated angioedema; (MMAE), Mental Composite Summary; (MCS), Minimal clinically important difference; (MCID), Number of days affected by angioedema; (NDAE), The Patient's global assessment of disease activity-visual analog scale; (PatGA-DA-VAS), Patients' global self-assessment of disease activity-verbal rating scale; (PatGA-DA-VRS), Patients' global self-assessment of quality of life; (PatGA-QoL), Physical Composite Summary (PCS), patient-reported outcome measures; (PROM), recurrent angioedema; (RAE), Smallest detectable change (SDC), Standard deviation; (sd), The 12-Item Short Form Health Survey; (SF-12), Urticaria Centers of Reference and Excellence; (UCARE), versus; (vs)

## Availability of data and materials

The datasets generated during the current study are available from the corresponding author on reasonable request.

## Funding

There was no external source of funding obtained. All expenses related to this research work were covered by the author.

## Ethics approval

The study was approved by ethical review board of Okmeydanı Training and Research Hospital (number of IRB: 580, 03.01.2017).

## Author contributions

EK, PKC, MM and KW designed the study and wrote the manuscript. EK, END, KK, PE, AG, SD, SB, EH, EBB and ÖA contributed to data collection, conception and design of the study, drafted the article and critical revision of the manuscript. EK, PKC, MM and KW performed the statistical analysis and interpretation of the results. All authors read and approved the final manuscript.

## Declaration of competing interest

Emek Kocaturk reports advisory board fees from 10.13039/100004336Novartis, and has served as a medical advisor for 10.13039/100004326Bayer, Menarini and 10.13039/100004339Sanofi. Marcus Maurer is or recently was a speaker and/or advisor for and/or has received research funding from Allakos, 10.13039/100002429Amgen, Aralez, ArgenX, Astra Zeneca, 10.13039/100012431Celldex, Centogene, 10.13039/100008322CSL Behring, 10.13039/100016525FAES, 10.13039/100004328Genentech, GIInnovation, Innate Pharma, 10.13039/100016348Kyowa Kirin, Leo Pharma, 10.13039/100004312Lilly, Menarini, Moxie, 10.13039/100004336Novartis, 10.13039/100004337Roche, 10.13039/100004339Sanofi/10.13039/100009857Regeneron, Third HarmonicBio, 10.13039/100011110UCB, and Uriach. Karsten Weller is or recently was a speaker and/or advisor for and/or has received research funding from Biocryst, 10.13039/100008322CSL Behring, Moxie, 10.13039/100004336Novartis, Shire/10.13039/100007723Takeda, and Uriach. The other authors have no conflict of interest to declare.
